# Carbapenem‐Resistant Gram‐Negative Bacilli in Mauritania: A Major Public Health Threat

**DOI:** 10.1002/mbo3.70337

**Published:** 2026-06-25

**Authors:** Fatimetou Ahmed Sid'Ahmed, Aminetou Mohamed Abbe, Mohamed Lemine Salem

**Affiliations:** ^1^ Laboratories of the Centre Hospitalier National de Nouakchott Mauritania; ^2^ Faculty of Sciences and Technology University of Nouakchott Mauritania; ^3^ Faculty of Medicine, Pharmacy and Odontostomatology University of Nouakchott Mauritania

**Keywords:** *Acinetobacter baumannii*, carbapenems, enterobacteriaceae, epidemiology, *Pseudomonas aeruginosa*, resistance

## Abstract

Carbapenem‐resistant Gram‐negative bacilli (CR‐GNB) are an emerging public health threat due to limited treatment options. This retrospective study investigated the epidemiology and resistance patterns of CR‐GNB isolated at the National Hospital Center of Nouakchott, Mauritania, between January 2020 and June 2022. Among 565 Gram‐negative bacilli isolates, 25 were carbapenem‐resistant, corresponding to a prevalence of 4.42%. *Klebsiella pneumoniae* and *Escherichia coli* were the most frequently identified species, and most isolates were recovered from urine samples. High resistance rates were observed to beta‐lactams and fluoroquinolones, whereas colistin, tigecycline, and amikacin retained good activity. Although the prevalence remains relatively low, continuous surveillance and appropriate infection control and antimicrobial stewardship measures are required to prevent the spread of carbapenem resistance.

## Graphical abstract

Carbapenem‐resistant Gram‐negative bacilli remain uncommon (4.42%) in Mauritania but are dominated by Klebsiella pneumoniae and Escherichia coli. Preserved activity of colistin, tigecycline, and amikacin highlights a narrowing therapeutic window and the urgent need for sustained surveillance and antimicrobial stewardship.

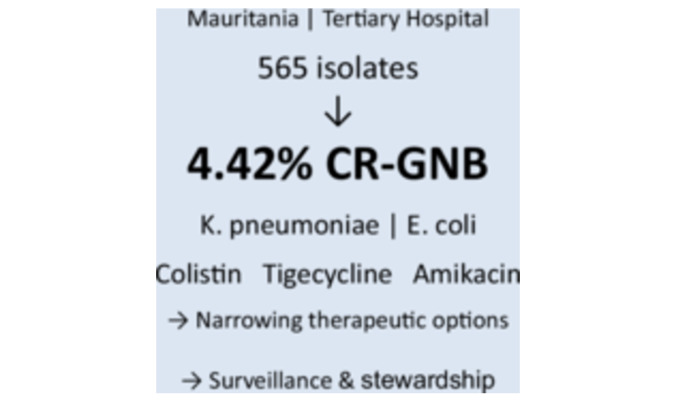

## 1 Introduction

Gram‐negative bacilli (GNBs) represent a heterogeneous group of bacteria, including fermentative species such as *Enterobacteriaceae* and non‐fermentative species such as *Pseudomonas aeruginosa* and *Acinetobacter baumannii*.

Enterobacteriaceae are among the most frequently isolated GNB in human infections, and constitute a major of both community‐acquired and nosocomial infections. The species most frequently encountered in clinical bacteriology are Escherichia, Klebsiella‐Enterobacter‐Serratia (KES), Proteus, Providencia, Salmonella‐Shigella (SS), and Yersinia. Pseudomonas aeruginosa and Acinetobacter baumannii are opportunistic pathogens often responsible for severe, potentially fatal nosocomial infections, particularly in intensive care units (Brink 2019; Baba Ahmed‐Kazi Tani and Arlet 2014; Rossolini et al. 2007).

Beta‐lactams are a family of antibiotics classified, based on their chemical structure, into four distinct groups: penicillins, cephalosporins, monobactams, and carbapenems. They are the most widely used antibiotics worldwide (Baba Ahmed‐Kazi Tani and Arlet 2014; Grall et al. 2011; Livermore and Woodford 2006; Wolff et al. 2009). However, these antibiotics have been exposed to the problem of resistance since they were first prescribed in the 1940s (Grall et al. 2011). Among them, carbapenems are considered last‐resort antibiotics and exhibit the broadest spectrum of activity, including against GNBs (Wolff et al. 2009).

These molecules are used exclusively in hospital setting and are reserved for the treatment of infections caused by multidrug‐resistant GNB (Nordmann and Poirel 2019; Ouedraogo et al. 2017). Like other beta‐lactams, carbapenems also act on the bacterial cell wall by inhibiting its synthesis through binding to penicillin‐binding proteins (PBPs) (Grall et al. 2011). Their antibacterial efficacy is attributed to their rapid penetration across bacterial membranes and their stability against most natural or acquired cephalosporinase as well as extended‐spectrum beta‐lactamases (ESBLs) (Grall et al. 2011; Naas T. Mechanisms of carbapenem resistance in GNBs and worldwide distribution).

Carbapenems are broad‐spectrum antibiotics that are highly effective against GNB, with the exception of ertapenem, which lacks activity against *Pseudomonas aeruginosa* and *Acinetobacter baumannii* (Grall et al. 2011). However, GNBscan develop resistance through various mechanisms. In particular, resistance mediated by the production of carbapenemases represents a major threat due to its rapid dissemination (Elshamy and Aboshanab 2020; Nordmann and Carrer 2010). In response, the WHO has launched several initiatives, including GLASS, GARDP, and IACG (https://www.who.int/fr/news-room/fact-sheets/detail/antibiotic-resistance) and in 2017 classified certain resistant strains as critical priorities (WHO, 2017). This resistance, which compromises the effectiveness of last‐resort antibiotics against multidrug‐resistant bacteria (MDR), is an urgent public health issue requiring urgent action to limit its spread and reinforce the available therapeutic arsenal (Brink 2019; Nordmann and Poirel 2019; Sleiman et al. 2021).

However, data on the epidemiology and resistance mechanisms of carbapenem‐resistant GNBs remain limited in low‐resource settings, including Mauritania. To the best of our knowledge, this study is among the first to provide a comprehensive description of the distribution of GNB species, their antimicrobial resistance profiles, and carbapenemase screening patterns within a hospital setting in Mauritania. The aim of this study was to describe the epidemiological characteristics and antimicrobial resistance profiles of carbapenem‐resistant Gram‐negative bacilli (CR‐GNB) isolated at the National Hospital Center of Nouakchott.

## 2 Materials and Methods

### 2.1 Study Design and Setting

This retrospective observational study was conducted at the central laboratory of the Center Hospitalier National de Nouakchott (CHN), the main referral hospital in Mauritania receiving both inpatients and outpatients. It was based on the analysis of bacteriological data collected between January 1, 2020, and June 30, 2022.

### 2.2 Inclusion and Exclusion Criteria

All GNB, including *Enterobacteriaceae, Pseudomonas aeruginosa, and Acinetobacter baumannii*) isolated from clinical samples processed in the laboratory during the study period were included.

Duplicate isolates from the same patient and the same anatomical site were excluded, as well as incomplete or poor‐quality samples.

### 2.3 Endpoint

Carbapenem resistance in GNB was defined according to EUCAST guidelines as resistance to at least one carbapenem.

### 2.4 Samples Collection and Data Sources

Urine samples were collected from patients in various inpatient and outpatient departments of the CHN. Patient data are anonymized to ensure confidentiality.

### 2.5 Microbiological Methods

‐Bacterial identification:

All GNB isolates were identified using standard biochemical techniques. Primary identification was performed using API galleries (API 20E for Enterobacteriaceae; bioMérieux, France), which assess metabolic profiles through a series of enzymatic reactions. The results were confirmed using the VITEK‐2 automated system (bioMérieux, France) when necessary. Only non‐duplicate isolates per patient were included in the analysis.

‐Antimicrobial Susceptibility Testing (AST):

Susceptibility testing was performed using the disk diffusion method on Mueller‐Hinton agar, following EUCAST guidelines. Standardized inocula were prepared by suspending 3–5 well‐isolated colonies in sterile saline to match a 0.5 McFarland turbidity standard. Sterile swabs were used to evenly inoculate the agar plates. Antibiotic disks were applied within 15 min of inoculation, and plates were incubated at 35 ± 1°C for 16–18 h under ambient air.

The tested antibiotics included beta‐lactams (penicillins, cephalosporins, carbapenems), aminoglycosides, fluoroquinolones, colistin, and tigecycline.

The carbapenems tested included imipenem, meropenem, and ertapenem.

Carbapenem resistance was defined according to EUCAST: isolates with reduced susceptibility to imipenem, meropenem, or ertapenem were classified as carbapenem‐resistant.

### 2.6 Statistical Analysis

Data were analyzed using Microsoft Excel 2016. Descriptive statistics were used to summarize the data.

### Ethics

The study was conducted using anonymized data. “Ethical approval was obtained from Health Research Ethics Committee (reference number: 008‐2025).”

## 3 Results

A total of 565 GNB isolates were collected during the study period. Among them, 25 were resistant to at least one carbapenem, corresponding to an overall prevalence of 4.42%. Additionally, 29 duplicate isolates were excluded Table 1.

**Table 1:** Distribution of GNB isolates and carbapenem resistance (January 2020‐June 2022).

Period	Number of isolates	Carbapenem resistant	Resistance%
2020	215	9	4.19%
2021	239	10	4.18%
January – June 2022	111	6	5.41%
Total (2020–2022)	565	25	4.42%

**Figure 1:**
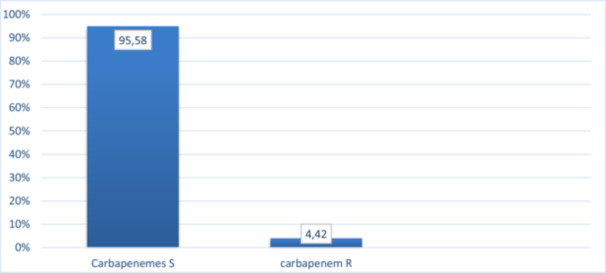
Frequency of isolation of carbapenem resistance in isolated GNB.

The distribution of carbapenem‐resistant GNB (CR‐GNB) isolates is presented in Figure 1.

### 3.1 Distribution of GNB Isolates by Sample Type

The majority of GNB were obtained from urine samples, representing 88.67% (*n* = 501) of all specimens. The distribution of isolates according to sample type is detailed in Table 2.

**Table 2:** Number and percentage of GNB isolates by sample type.

Withdrawal	Number	Percentage (%)
ECBU	501	88.67
Deeper	27	4.78
PV	16	2.84
Ear pus	14	2.49
Puncture fluids	05	0.88
Sputum	01	0.17
Blood cultures	01	0.17
Total	565	100

### 3.2 Distribution of GNB Isolates by Species

Among the identified isolates, *Escherichia coli* was the predominant species, accounting for 63.89% (*n* = 361) of all GNB isolates. The overall species distribution is summarized in Table 3.

**Table 3:** Number and percentage of GNB isolates by species.

Germ	Number	Percentage (%)
*Escherichia coli*	361	63.89
*Klebsiella pneumonia*	80	14.16
*Pseudomonas aeruginosa*	27	4.78
*Klebsiella oxytoca*	25	4.42
*Klebsiella aerogenes*	20	3.54
*Enterobacter cloacae*	15	2.65
*Proteus mirabilis*	15	2.65
*Acinetobacter baumannii*	06	1.06
Serratia spp	05	0.88
Citrobacter spp	04	0.70
Salmonella spp	04	0.70
*Proteus penneri*	02	0.40
*Providencia rettgeri*	01	0.17
Total	565	100

### 3.3 Distribution of Carbapenem‐Resistant GNB Isolates by Species

**Figure 2:**
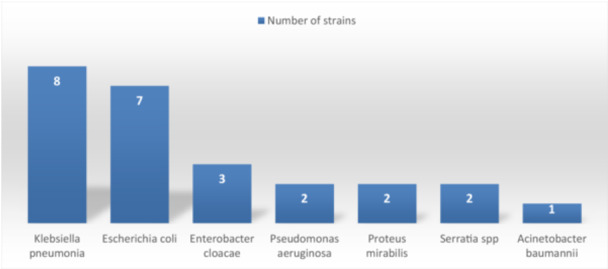
Number of carbapenem‐resistant strains by species. NB: the *Acinetobacter baumannii* strain isolated was resistant to imipenem.

Among the 25 carbapenem‐resistant (CR‐GNB), *Klebsiella pneumonia* was the most frequently identified species (32%), followed by *Escherichia coli* (28%). The species distribution of BNB‐RC isolates is presented in Figure 2.

### 3.4 Distribution of Carbapenem‐Resistant GNB Isolates by Sample Type

Most carbapenem‐resistant isolates were recovered from urine samples, accounting for 84% (*n* = 21). The distribution by sample type is provided in Table 4.

**Table 4:** Number and percentage of carbapenem‐resistant GNB isolates by sample type.

Withdrawal	Number	Percentage (%)
ECBU	21	84
Ear pus	02	8
Deeper	01	4
Puncturefluid	01	4
Total	25	100

### 3.5 Demographic Characteristics of Patients With CR‐GNB Isolates

**Figure 3:**
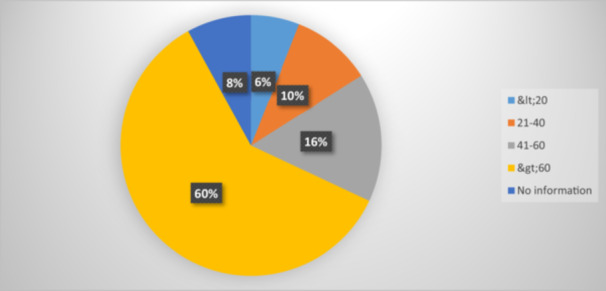
Percentage of RC‐GNB isolates by age.

Age distribution: The mean age of patients was 42.9 years, with a range from 1 year and 8 months to 75 years. The age distribution is shown in Figure 3.

**Figure 4:**
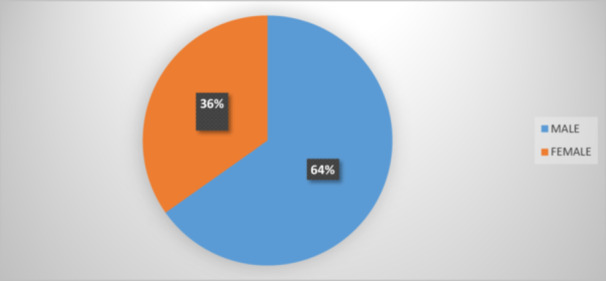
Frequency of RC‐GNB strains by gender.

Gender distribution: Male patients were more frequently affected, accounting for 64% (*n* = 16) of cases, with a male‐to‐female ratio of 1.8. The gender distribution is illustrated in Figure 4.

### 3.6 Distribution of CR‐GNB Isolates by Hospital Department

The CR‐GNB isolates originated from various inpatient and outpatient departments of the CHN, as well as from other healthcare facilities. Their distribution of by department is presented in Table 5.

**Table 5:** Number and percentage of carbapenem‐resistant GNB strains by department.

Service	Number	Percentage (%)
Urology	09	36
Nephrology	06	24
Resuscitation	05	20
Cardiology	01	4
PediatricSurgery	01	4
No information	03	12
Total	25	100

### 3.7 Clinical Characteristics of Patients

The clinical characteristics observed among patients with CR‐GNB are presented in Table 6.

**Table 6:** Number and percentage of carbapenem‐resistant GNB strains according to patients' clinical characteristics.

Risk factors	Number	Percentage (%)
Diabetes	08	32
Diabetes+HBP	05	20
IRCT	04	16
CKD + HTA	03	12
HTA	03	12
Circumcision	01	4
WTO	01	4
Total	25	100

### 3.8 Antibiotic Resistance Profile of CR‐GNB Isolates

Resistance rates of CR‐GNB isolates to other antibiotic classes are detailed in Table 7.

**Table 7:** Resistance profile to other antibiotics in all RC‐GNB strains.

Antibiotic	Resistance %	Sensitivity
Amoxicillin	100	0
Amoxicillin + clavulanicacid	72	28
Ticarcillin	100	0
Piperacillin	100	0
Cephalotin	100	0
Cefoxitin	76	24
Cefotaxime/Ceftriaxone/Cefixime	80	20
Ceftazidime	80	20
Nalidixicacid	91	09
Ofloxacin	87	13
Ciprofloxacin	80	20
Levofloxacin	74	26
Amikacin	24	76
Gentamicin	82	18
Tobramycin	67	33
Sulfamethoxazole+trimethoprim	90	10
Colistin (other than Proteus mirabilis and Serratia spp)	0	100
Fosfomycin	47	53
Nitrofurans	87	13
Chloranphenicol	76	24
Tigecycline	15	85

## 4 Discussion

Emerging antibiotic resistance among GNB represents a major global public health concern. Carbapenem‐resistant GNB (CR‐GNB), in particular, are associated with limited therapeutic options and increased mortality, especially in hospital settings (Breijyeh et al. 2020; Jean et al. 2022; Mairi et al. 2018; Theuretzbacher 2017; Doi et al. 2017; Logan and Weinstein 2017a).

Carbapenem resistance is mainly driven by the production of carbapenemases and/or associated mechanisms such as porin loss and efflux pump overexpression (Stahl JP, Boutoille D, Saidani N, et al. CARBAR study in France: epidemiology of Gram‐negative pathogens). Among *Enterobacteriaceae*, *Klebsiella pneumoniae,* and *Escherichia coli* are the most frequently implicated species in hospital‐associated infections and are major reservoirs of resistance genes (Wyres et al. 2020; Effah et al. 2020).

In the present study, the overall prevalence of CR‐GNB was 4.42%. Similar rates have been reported in Lebanon (4.75%) (Hamze 2018) and the United Arab Emirates (4.6%) (Moubareck et al. 2018), while higher rates have been described in some European settings (Stahl JP, Boutoille D, Saidani N, et al. CARBAR study in France: epidemiology of Gram‐negative pathogens). These findings highlight the growing dissemination of carbapenem resistance across different geographical regions.

Our results showed a predominance of Enterobacteriaceae among CR‐GNB isolates, particularly Klebsiella pneumoniae, followed by Escherichia coli. This predominance has also been reported in several studies (Stahl et al. 2020; Saeed et al. 2019; Zhang et al. 2018; Benammar et al. 2017; Satlin et al. 2017) and may be explained by their intestinal reservoir, high bacterial load, and capacity for horizontal gene transfer (Peri et al. 2019).

Urinary samples accounted for the majority of isolates, and most CR‐GNB were recovered from urology‐related specimens. This may reflect the high frequency of urinary tract infections and the use of invasive urinary devices in hospitalized patients (Hamze 2018; Lemine Ould Salem 2022).

The mean age of patients was 42.9 years, and males were more frequently affected. This male predominance has been reported in other studies and may be related to a higher exposure to healthcare procedures in certain clinical conditions (Hamze 2018; Saeed et al. 2019).

CR‐GNB isolates demonstrated high levels of resistance to most beta‐lactams and fluoroquinolones, leaving limited therapeutic options. Colistin, tigecycline, and fosfomycin remained the most active agents (Hamze 2018; Saeed et al. 2019; Chew et al. 2017; Khare et al. 2017). However, the emergence of multidrug resistance significantly restricts therapeutic strategies and highlights the need for strict antibiotic stewardship programs (World Health Organization 2017; Magiorakos et al. 2017; French et al. 2017; Logan and Weinstein 2017b).

The present study has several limitations, including its retrospective design, incomplete clinical data, and the relatively small number of CR‐GNB isolates. In addition, the single‐center nature of the study limits the generalizability of the findings. The relatively small number of carbapenem‐resistant isolates (*n* = 25) limits the generalizability of our findings.

### 4.1 Study Biases and Limitations

This study has several limitations should be considered when interpreting the results. Firstly, it is a retrospective study, which may induce selection bias due to the absence of randomization and dependence on data already available in the medical records. In addition, some clinical data were incomplete, particularly regarding prior antibiotic therapy, length of hospital stay and use of invasive medical devices, which are important clinical characteristics for MDR infections.

Second, the relatively small number of carbapenem‐resistant isolates (n = 25) limits the statistical power of the analysis and may underestimate the diversity of resistance mechanisms circulating in Mauritania.

Furthermore, as the study was carried out in a single hospital, it does not necessarily reflect the epidemiological situation in other health facilities in the country, introducing a representativeness bias.

Despite these limitations, this study provides one of the first insights into carbapenem resistance and underscores the urgent need for strengthened microbiological surveillance and antimicrobial stewardship strategies.

## 5 Conclusion and Recommendations

This study described the epidemiological profile of carbapenem resistance in GNB isolated at the central laboratory of the Center Hospitalier National de Nouakchott between January 2020 and June 2022. The prevalence of resistance observed in the GNB studied was 4.42%, indicating a worrying trend towards the spread of such resistance. The high levels of multidrug resistance identified among the isolates significantly limit available therapeutic options and reinforce the global concern regarding antimicrobial resistance as a major public health threat.

In light of these findings, several measures should be prioritized. Routine microbiological testing should be encouraged prior to the initiation of empirical antibiotic therapy whenever clinically feasible. Strengthening the detection, reporting, and infection control management of multidrug‐resistant organisms is also essential, alongside strict adherence to hospital hygiene protocols.

Healthcare establishments, for their part, need to consolidate their preventive measures, in particular by training staff and activating the Comités de Lutte contre les Infections Nosocomiales (Nosocomial Infection Control Committees).

Health authorities need to step up awareness‐raising campaigns on the rational prescription of antibiotics, and ensure strict control of their use, including in the veterinary and agri‐food sectors. Finally, involving the general public in the fight against antibiotics requires health education aimed at limiting self‐medication and promoting compliance with medical prescriptions.

In conclusion, controlling the spread of carbapenem‐resistant GNBs requires a coordinated response, integrating surveillance, prevention, rationalization of antibiotic therapy and mobilization of all players in the healthcare system.

In conclusion, controlling the spread of carbapenem‐resistant GNB requires a coordinated and multidisciplinary approach integrating surveillance, infection prevention, antimicrobial stewardship, and public health interventions.

## Author Contributions

**Fatimetou Ahmed Sid'Ahmed:** conceptualization, writing – original draft, methodology, validation, visualization, investigation, funding acquisition, software, formal analysis, project administration, data curation. **Aminetou Mohamed Abbe:** visualization, validation, writing – review and editing, formal analysis, supervision. **Mohamed Lemine Salem:** validation, visualization, writing – review and editing, supervision, software, formal analysis, writing – original draft.

## Funding

The authors have nothing to report.

## Ethics Statement

The authors declare that the work described in the article has been carried out in compliance with ethical principles and current recommendations.

## Conflicts of Interest

The authors declare that they have no ties of interest. All authors have read and approved the final version of the article.

## Data Availability

The data that support the findings of this study are available on request from the corresponding author. The data are not publicly available due to privacy or ethical restrictions.
